# Laser printing: trends and perspectives

**DOI:** 10.1007/s00339-022-06158-9

**Published:** 2022-10-31

**Authors:** Boris Chichkov

**Affiliations:** Institute of Quantum Optics, Leibniz University Hannover, Welfengarten 1, 30167 Hannover, Germany

**Keywords:** Laser printing, Nanoparticles, Bioprinting, Additive manufacturing

## Abstract

In this paper, I present my personal view on the possible development and applications of laser printing technologies based on laser-induced forward transfer of inorganic and biological materials. Laser printing of micro- and nanoparticles, living cells, and microorganisms are discussed.

## Introduction

This paper is written following a personal invitation from Prof. Dr. Thomas Lippert, Editor-in-Chief of Applied Physics A, in celebration of the 50th anniversary of this journal. According to the fact, that our paper, published in 1996 in Applied Physics A, and discussing femtosecond, picosecond and nanosecond laser ablation of solids [1] became a most cited paper in 50 years history of this journal, I and my colleagues were invited to write about our current research. This is a great honor for me, which allows presenting my personal view on the development of laser printing technologies based on laser-induced forward transfer of materials in molten or liquid phases. Different aspects of laser printing have been recently reviewed, including laser micro- and nanoprinting [2], laser printing of living cells [3–5], and laser printing of microorganisms [6]. In this paper, I will focus on perspectives and possible trends in the development of laser printing technologies.

## Laser printing of micro- and nanoparticles

**Fig. 1:**
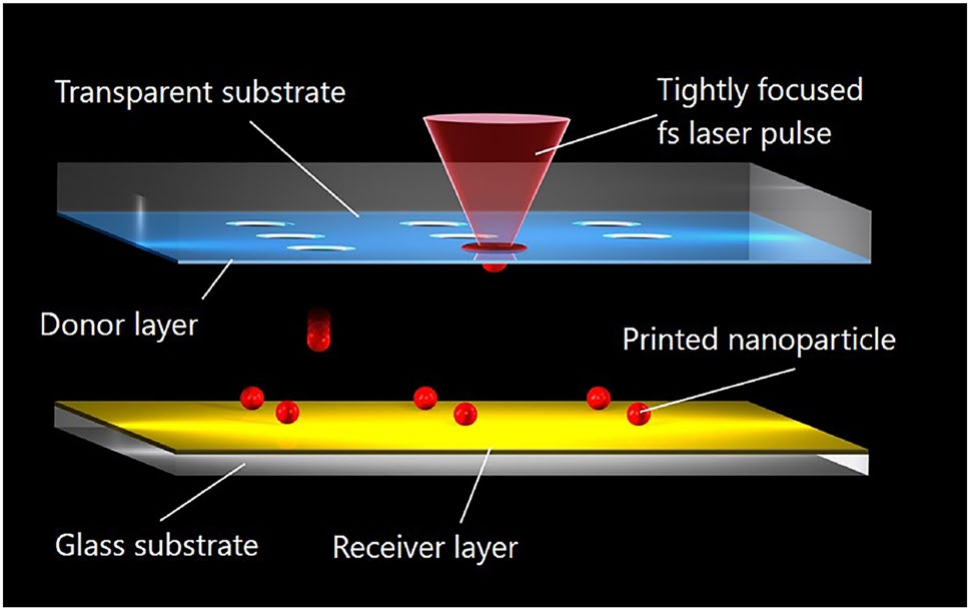
Schematic setup for laser printing of nanoparticles

For laser printing of nanoparticles the following setup shown in Fig. 1 is applied. A thin layer of a donor material to be printed (shown by blue color) is coated on a transparent substrate. Femtosecond laser pulses are focused onto this layer, heat it up and transfer material into a molten nanodroplet flying towards the receiver substrate. Depending on the distance between the donor and receiver substrates very precise positioning of nanodroplets in from of nanoparticles is possible. Laser printing allows the generation of spherical nanoparticles with controllable sizes and provides a powerful method for the arrangement of nanoparticles in a very precise manner. The size (radius $r_n$) of the printed nanoparticles can be estimated by equating the volumes of the molten material and of the spherical nanoparticle, $r_n=(3r_m^2h/4)^{1/3}$, where $r_m$ is the radius of the molten area, assumed for estimates to be approximately equal to the focal spot radius, and *h* is the thickness of the donor layer, which is usually in the range of 50–100 nanometers. The radius of the molten area depends on the laser pulse energy. Therefore, the size of the printed nanoparticles can be varied by changing the laser pulse energy. Physical mechanisms behind the generation of nanoparticles have been experimentally studied and discussed in [7]. At present, laser printing has been applied to the controlled fabrication of metallic (Au, Ag, Al, Cu, Fe, etc.) and semiconductor (Si, Ge, etc.) nanoparticles with precisely adjustable radii between 50 nm and 1 $\mu$m and their accurate positioning on a desired substrate.

**Fig. 2:**
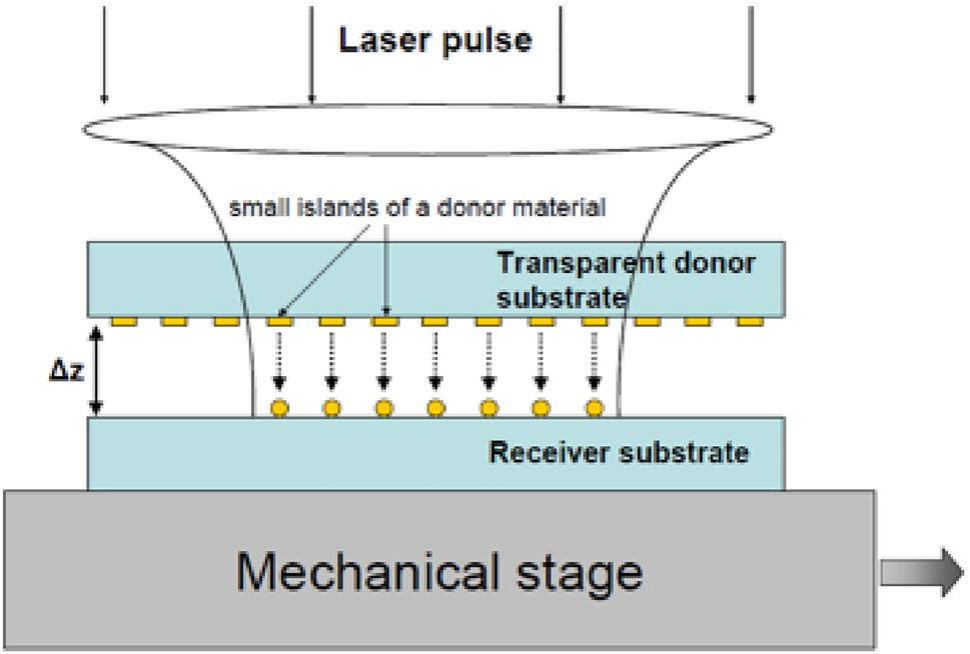
Generation of nanoparticles from lithographically pre-patterned film

The donor material layer can be lithographically pre-patterned to produce separated nanostructures, e.g., nano-islands with the radius $r_i$. In this case much smaller nanoparticles with $r_n=(3r_i^2h/4)^{1/3}$ can be generated. Moreover, many nanoparticles can be produced by a single laser pulse as it is schematically shown in Fig. 2.

The laser-printed nanoparticles can be applied as metasurfaces with desired optical properties [8, 9], optical sensors [10], optical couplers for light manipulation [11], etc. Since optical applications of laser printed nanoparticles are well documented, in this paper we discuss other potential application areas which have not been addressed so far.

**Fig. 3:**
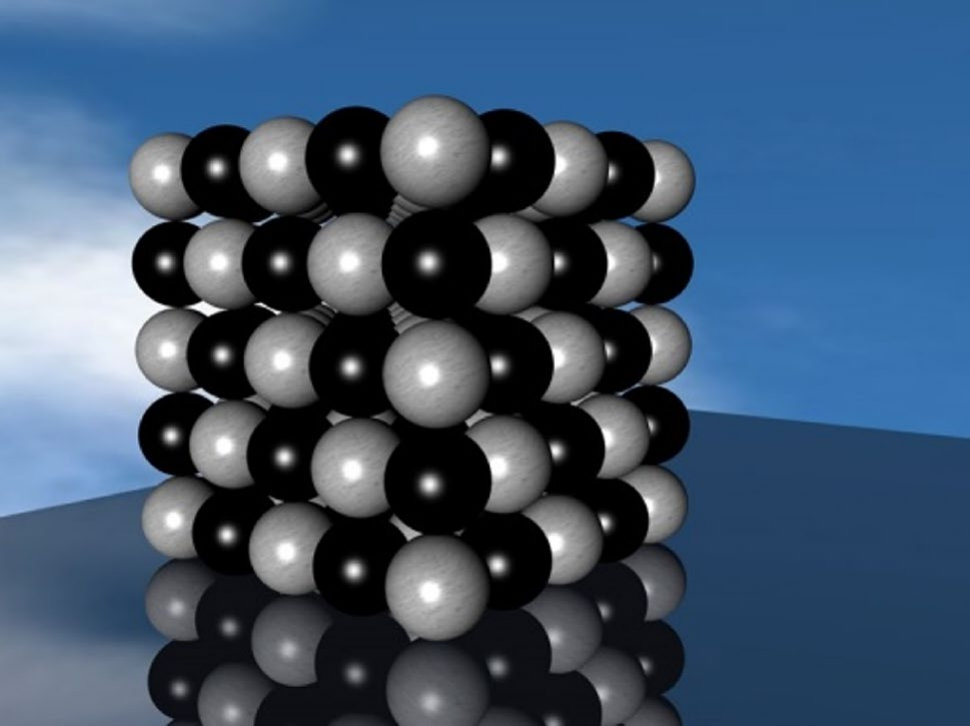
Illustration of a possible design of a laser printed composite nanomaterial

One of interesting and promising applications of laser printing technology could be nano-assembly of novel materials. Printing of nanoparticles consisting of different materials layer-by-layer can produce novel composite multi-functional materials with unique combination of desired properties (e.g., conductive, magnetic, transparent, etc.). Schematic illustration of a possible design of laser printed nanomaterial is shown in Fig. 3. By heat treatment, all nanoparticles can be molten together to form a new solid. This approach can be considered as laser-based additive micro- and nanomanufacturing of novel materials. With modern femtosecond laser systems operating at a 1 MHz repetition rate one can print up to 1 million particles per second, therefore, the discussed above idea of nano-assembly of novel materials can become practicable for the production of small material samples.

**Fig. 4:**
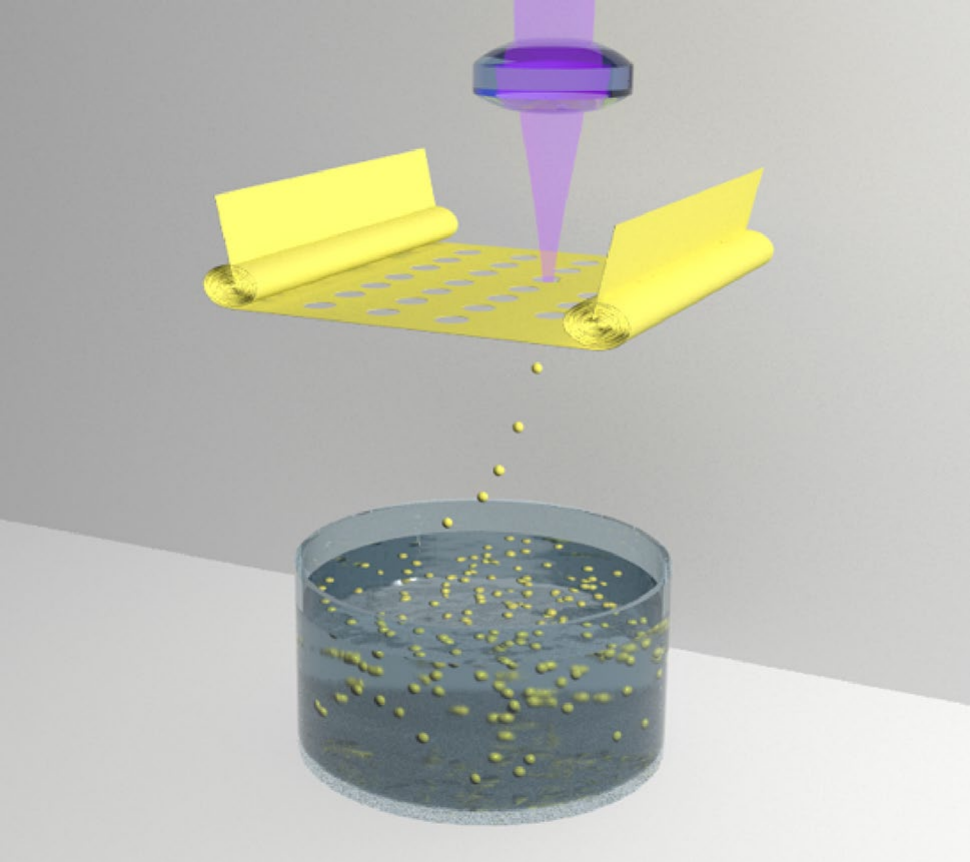
Schematic setup for the generation of mono-dispersed colloidal microparticles

Another potential application of laser printing technology could be the generation of mono-dispersed colloidal micro- and nanoparticles. Microparticles can be printed not only using the setup shown in Fig. 1. They can be printed from thin foils. The corresponding setup for the printing of mono-dispersed metal microparticles is illustrated in Fig. 4. For printing of microparticles from thin foils higher laser pulse energies and/or longer laser pulses, compared to femtosecond laser printing shown in Fig. 1, will be required. Laser pulse energy should be high enough to drive the thermal wave and the molten material front through the foil. The molten material will be propelled in the forward direction due to the reactive recoil pressure of the plasma plume and evaporated material at the front side. Note that laser printing of colloidal microparticles become practicable with high repetition rate (up to 1 MHz) lasers, allowing the generation of up to 1 million micro- or nanoparticles per second. This method has distinct advantages compared to chemical synthesis and methods based on laser ablation and laser fragmentation.

Laser printing of micro- and nanoscale particles can also find applications in additive manufacturing of 3D structures (3D printing). Usually, for laser-based additive manufacturing material micropowders available on the market are used. The dominant form of 3D printing is powder bed fusion, in which laser beam, in case of selective laser melting (SLM), fuses 20–100 $\mu$m powder particles layer by layer until a required object is generated [12]. One can combine SLM with powder injection deposition which is used in the method called laser-engineered net shaping (LENS) [13]. Both methods are cost effective, work well with a wide variety of metals and alloys (including stainless steel, aluminum, nickel, cobalt-chrome, and titanium alloys), produce an excellent surface finish, and are industrially safe.

**Fig. 5:**
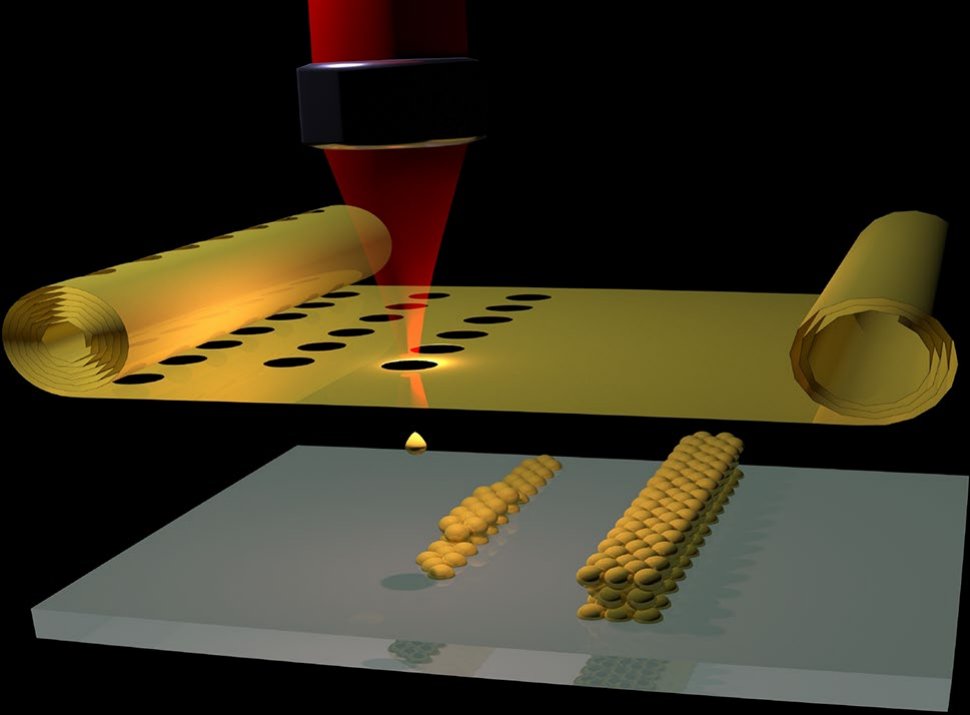
Laser printing of particles-on-demand for powder-free additive manufacturing

Instead of using a powder bed, one can develop a new particle-on-demand generation method using high-speed laser printing of microparticles, placing them at the desired positions and fusing them later analogous to the SLM procedure to generate 3D objects (Fig. 5). At the printing speed of 1 million particles per second, by printing 10 $\mu$m diameter particles in close contact with each other in a single-layer square structure, a highly ordered 1 x 1 cm size microparticle-array can be generated in 1 second. By laser beam scanning and independent movement of foil and receiver substrates, particles can be printed on top of each other producing the desired 3D structures. This will be an energy-efficient, powder-free, safe-handling technology, where particles will be placed on demand and either sintered or fused together using another or the same laser system. These procedures could be referred to either as Laser Printing and Sintering (LPS) or Laser Printing and Melting (LPM). Powder-free additive manufacturing will allow to use this technology in space and in the absence of gravity.

## Laser printing of living cells

3D laser printing of living cells for the fabrication of biological tissues and organs is a very promising technique having many advantages: possibility to print small droplets with volumes down to 1 picoliter and materials with high and low viscosities; high printing resolution (< 10$\mu$m); high cell survival (up to 100%); high printed cell densities ($>10^8$ cells/ml) comparable with the cell density in living organs; and contamination-free bioprinting at 2.94 $\mu$m laser wavelength corresponding to the peak absorption in water. Bioprinting, without a special absorption layer, at 2.94 $\mu$m wavelength has been first demonstrated in [14]. Parameters of the commercial laser which we applied in this work were not optimal. More short nanosecond laser pulses and higher repetition rates are required.

In 2006, Shinya Yamanaka discovered possibilities for reprogramming of human somatic cells to a pluripotent embryonic-like stem cells, called induced pluripotent stem cells (iPSCs). Human iPSCs can be differentiated towards all functional cell types and returned to the patient without immunological concerns. This revolutionary technique offers interesting opportunities for the fabrication of replacement tissues and organs for personalized regenerative therapies or realization of organ-on-chip systems for diagnostics and drug development. Laser bioprinting of iPSCs has been demonstrated by us in [15] and the impact on cells’ behavior, pluripotency, and differentiation has been investigated.

In spite tremendous progress in biofabrication and tissue engineering, building 3D vascularised organs remains the major unsolved challenge to be overcome. We have recently observed that endothelial cells printed in a line form microcapillaries with a lumen on their own, and that endothelial and smooth muscle cells, printed in separate positions in one pattern, self-organise in vessel-like structures [16]. These results demonstrate that 3D microcapillary networks can be laser printed. In conclusion, I believe that laser bioprinting will be able to generate human tissues and organs from a variety of cell types with precise 3D architecture and life-sustaining vascular networks.

## Laser printing of microorganisms

The possibility to print small droplets with volumes down to 1 picoliter provides opportunities for the printing and selection of single microorganisms. The access to single microorganisms is very important since the majority of natural microbiomes remain uncultivated. The microbial diversity on Earth is impressively rich, but more than 99% of the potentially $10^{11}$ – $10^{12}$ microbial species remain undiscovered to date [17]. One can speak about this undiscovered part of microbiome as a “Dark living matter”. Note that approximately, $10^{14}$ microorganisms inhabit a single human and $10^{30}$ cells of bacteria and archaea are estimated to inhabit Earth [17].

Metagenomics may provide data on the organisms independent of the ability to culture them. Metagenomics is the study of total genomic DNA obtained directly from animal gut, soil, sea water, desert, etc. A review of limitations and opportunities in metagenomics one can find in [18].

Laser bioprinting of microorganisms allows getting access to non-cultivated microorganisms and single-cell genomics. It can help to overcome existing challenges with unculturable bacteria and preparation of pure cultures [19, 20].

At present, we are working on laser printing of oral microorganisms. The oral cavity has the second largest and diverse microbiota after the gut harboring over 700 species of bacteria [21]. From them 54% are officially named, 14% are unnamed (but cultivated) and 32% are uncultivated. Using laser bioprinting we aim to develop technology for patient-specific diagnostics of oral bacteria. This work is in progress and our results will be published elsewhere.

## Conclusion

In this paper, the potential and possible applications of laser printing technologies have been briefly reviewed. Laser printing of micro- and nanoparticles for assembly of novel materials, for generation of mono-dispersed colloids, for powder-free additive manufacturing have been discussed. I have also tried to share my enthusiasm with respect to further development and prospects of laser bioprinting of living cells and microorganisms. I hope that this paper will attract attention and motivate other scientists to join this field of research.

## References

[1] Chichkov BN, Momma C, Nolte S, von Alvensleben F, Tünnermann A (1996). Femtosecond, picosecond and nanosecond laser ablation of solids. Appl. Phys. A.

[2] Li Q, Grojo D, Alloncle A-P, Chichkov B, Delaporte P (2019). Digital laser micro- and nanoprinting. Nanophotonics.

[3] L. Koch, A. Deiwick, and B. Chichkov, Laser-based cell printing. in A. Ovsianikov et al. (eds.), 3D Printing and Biofabrication, Reference Series in Biomedical Engineering (Springer International Publishing AG) 303-328 (2018)

[4] Dou C, Perez V, Qu J, Tsin A, Xu B, Li J (2021). A State-of-the-Art Review of Laser-Assisted Bioprinting and its Future Research Trends. Chem. Bio. Eng. Rev..

[5] Yang H, Yang K-H, Narayan RJ, Ma S (2021). Laser-based bioprinting for multilayer cell patterning in tissue engineering and cancer research. Essays in Biochemistry.

[6] Cheptsov VS, Tsypina SI, Minaev NV, Yusupov VI, Chichkov BN (2019). New microorganism isolation techniques with emphasis on laser printing. Int. J. Bioprint..

[7] Kuznetsov AI, Unger C, Koch J, Chichkov BN (2012). Laser-induced jet formation and droplet ejection from thin metal films. Appl. Phys. A.

[8] U. Zywietz, T. Fischer, A. Evlyukhin, C. Reinhardt, B. Chichkov, Laser Printing of Nanoparticles. Book Chapter in Laser Printing of Functional Materials: 3D Microfabrication, Electronics and Biomedicine, (Wiley-VCH Verlag GmbH & Co. KGaA Weinheim, Germany), 251-268, (2018)

[9] Evlyukhin AB, Matiushechkina M, Zeninm VA, Heurs M (2020). B, N, Chichkov, Lightweight metasurface mirror of silicon nanospheres [Invited]. Optical Materials Express.

[10] Aristov AI, Zywietz U, Evlyukhin AB, Reinhardt C, Chichkov BN, Kabashin AV (2014). Laser-ablative engineering of phase singularities in plasmonic metamaterial arrays for biosensing applications. Appl. Phys. Lett..

[11] Gulkin DN, Popkova AA, Afinogenov BI, Shilkin DA, Kuršelis K, Chichkov BN, Bessonov VO, Fedyanin AA (2021). Mie-driven directional nanocoupler for Bloch surface wave photonic platform. Nanophotonics.

[12] Yap CY, Chua CK, Dong ZL, Liu ZH, Zhang DQ, Loh LE, Sing SL (2015). Review of selective laser melting: Materials and applications. Applied Physics Reviews.

[13] B. Zheng, N. Yang, J. Yee, K. Gaiser, W. Y. Lu, L. Clemon, Y. Zhou, E. J. Lavernia, J. M. Schoenung, Review on laser powder injection additive manufacturing of novel alloys and composites, Proc. SPIE 9738, Laser 3D Manufacturing III, 97380O (2016)

[14] Sorkio A, Koch L, Koivusalo L, Deiwick A, Miettinen S, Chichkov B, Skottman H (2018). Human stem cell based corneal tissue mimicking structures using laser-assisted 3D bioprinting and functional bioinks. Biomaterials.

[15] L. Koch, A. Deiwick, A. Franke, K. Schwanke, A. Haverich1, R. Zweigerdt, B. Chichkov, Laser bioprinting of human induced pluripotent stem cells–the effect of printing and biomaterials on cell survival, pluripotency, and differentiation. Biofabrication **10**, 035005 (2018) 10.1088/1758-5090/aab981 29578448

[16] Koch L, Deiwick A, Chichkov B (2021). Capillary-Like Formations of Endothelial Cells in Defined Patterns Generated by Laser Bioprinting. Micromachines.

[17] Loceya KJ, Lennon JT (2016). Scaling laws predict global microbial diversity. PNAS.

[18] Quince C, Walker AW, Simpson JT, Loman NJ, Segata N (2017). Shotgun metagenomics, from sampling to analysis. Nature Biotechnology.

[19] Austin B (2017). The value of cultures to modern microbiology. Antonie van Leeuwenhoek.

[20] Bodor A (2020). Challenges of unculturable bacteria: environmental perspectives. Rev. Environ. Sci. Biotechnol..

[21] Deo PN, Deshmukh R (2019). Oral microbiome: Unveiling the fundamentals. Journal of Oral and Maxillofacial Pathology.

